# Comparative evaluation of shape retrieval methods on macromolecular surfaces: an application of computer vision methods in structural bioinformatics

**DOI:** 10.1093/bioinformatics/btab511

**Published:** 2021-07-11

**Authors:** Mohamed Machat, Florent Langenfeld, Daniela Craciun, Léa Sirugue, Taoufik Labib, Nathalie Lagarde, Maxime Maria, Matthieu Montes

**Affiliations:** Laboratoire GBCM, EA 7528, Conservatoire National des Arts et Métiers, Hesam Université, Paris 75003, France; Laboratoire GBCM, EA 7528, Conservatoire National des Arts et Métiers, Hesam Université, Paris 75003, France; Laboratoire GBCM, EA 7528, Conservatoire National des Arts et Métiers, Hesam Université, Paris 75003, France; Laboratoire GBCM, EA 7528, Conservatoire National des Arts et Métiers, Hesam Université, Paris 75003, France; Laboratoire GBCM, EA 7528, Conservatoire National des Arts et Métiers, Hesam Université, Paris 75003, France; Laboratoire GBCM, EA 7528, Conservatoire National des Arts et Métiers, Hesam Université, Paris 75003, France; Laboratoire GBCM, EA 7528, Conservatoire National des Arts et Métiers, Hesam Université, Paris 75003, France; Laboratoire XLIM, UMR CNRS 7252, Université de Limoges, Limoges 87000, France; Laboratoire GBCM, EA 7528, Conservatoire National des Arts et Métiers, Hesam Université, Paris 75003, France

## Abstract

**Motivation:**

The investigation of the structure of biological systems at the molecular level gives insights about their functions and dynamics. Shape and surface of biomolecules are fundamental to molecular recognition events. Characterizing their geometry can lead to more adequate predictions of their interactions. In the present work, we assess the performance of reference shape retrieval methods from the computer vision community on protein shapes.

**Results:**

Shape retrieval methods are efficient in identifying orthologous proteins and tracking large conformational changes. This work illustrates the interest for the protein surface shape as a higher-level representation of the protein structure that (i) abstracts the underlying protein sequence, structure or fold, (ii) allows the use of shape retrieval methods to screen large databases of protein structures to identify surficial homologs and possible interacting partners and (iii) opens an extension of the protein structure–function paradigm toward a protein structure-surface(s)-function paradigm.

**Availabilityand implementation:**

All data are available online at http://datasetmachat.drugdesign.fr.

**Supplementary information:**

Supplementary data are available at *Bioinformatics* online.

## 1 Introduction

Proteins are macromolecules involved in most biological processes. Protein structures have been classified based on their backbone conformation and evolutionary history (Chandonia *et al.*, 2019; Dawson *et al.*, 2017). Proteins interact through their molecular surface that is considered as a higher-level representation of the protein structure (Guzenko *et al.*, 2020; Han *et al.*, 2019; Pawlowski and Godzik, 2001). Molecular surface abstracts the underlying protein sequence, structure and fold into a continuous shape with geometric and chemical features that fingerprint their interactions (Gainza *et al.*, 2020; Shulman-Peleg *et al.*, 2004). Functionally related proteins often share similar molecular surface properties despite a potentially low sequence and/or backbone conformation similarity (Han *et al.*, 2019; Sael *et al.*, 2008a). Different categories of methods based on protein surface shape have been developed over time such as protein–protein docking (Ruiz Echartea *et al.*, 2019; Schneidman-Duhovny *et al.*, 2005), protein structure alignment (Mavridis and Ritchie, 2010) or protein surface shape comparison methods (Craciun *et al.*, 2017; Gainza *et al.*, 2020; Gao *et al.*, 2016; Gramada and Bourne, 2006; Guzenko *et al.*, 2020; Han *et al.*, 2019; Mak *et al.*, 2008; Sael *et al.*, 2008b). Shape comparison and retrieval methods have been extensively developed in the computer vision field, notably for military, civil security or medical imaging applications (Bustos *et al.*, 2007). These methods, that can be applied to protein surface shapes, can be classified into different categories according to their shape representation: (i) shape retrieval methods based on spectral geometry to establish a relationship between the surface shape and the spectra of the Laplace-Beltrami operator; a spectrum of the Laplace-Beltrami operator is a fingerprint composed of the eigenvalues obtained using the differential Laplace-Beltrami operator (Reuter *et al.*, 2006), (ii) shape retrieval methods based on histograms summarizing local or global geometrical features of the surface shape (Rusu *et al.*, 2010), (iii) shape retrieval methods based on molecular surface maps, i.e. the projection(s) of the protein topography in the 2D space (Papadakis *et al.*, 2010), (iv) shape retrieval methods based on the moments of 3D Zernike polynomials that best fit the molecular surface shape (La *et al.*, 2009), (v) shape retrieval methods based on geometric learning (Gainza *et al.*, 2020; Monti *et al.*, 2017).

The evaluation of the performance of shape retrieval methods in the literature is performed classically during the SHREC community benchmark (Veltkamp *et al.*, 2006) where joint efforts between the structural bioinformatics and the computer vision communities have been performed to develop benchmarking datasets on protein shapes (Langenfeld *et al.*, 2018; 2019; 2020; Mavridis *et al.*, 2010; Song *et al.*, 2017).

In the present work, we evaluate the performance of four different shape retrieval methods [3D-Surfer (La *et al.*, 2009), PANORAMA (Papadakis *et al.*, 2010), ShapeDNA (Reuter *et al.*, 2006) and VFH (Rusu *et al.*, 2010)] on the complete cross-comparison of the SHREC 2019 protein shapes benchmarking dataset (5298 shapes) (Langenfeld *et al.*, 2019). PANORAMA, ShapeDNA and VFH have shown top performance on non-protein shapes benchmarks (Li *et al.*, 2015; Lian *et al.*, 2013; 2015; Li and Hamza, 2014). As a reference, we include different protein structure comparison methods [CE (Shindyalov and Bourne, 1998), DeepAlign (Wang *et al.*, 2013), TM-Align (Zhang and Skolnick, 2005) and lDDT (Mariani *et al.*, 2013)]. We also illustrate the performance of these shape retrieval methods on calmodulin, a protein displaying large conformational changes. Finally, we highlight the ability of these methods to identify distant surficial homologs. This work illustrates the interest for the protein surface shape as a higher-level representation of the protein structure that abstracts the underlying protein sequence, structure or fold and allows the use of shape retrieval methods to screen large database of protein structures to identify surficial homologs and possible interacting partners.

## 2 Materials and methods

### 2.1 Datasets

**Set**  A has been designed for the evaluation of the performance of shape retrieval methods on protein shapes for the community benchmark SHREC 2019 (Langenfeld *et al.*, 2019). The dataset comprises 5298 experimental conformations of protein domains extracted from 211 PDB entries resolved by NMR. It is available at http://shrec2019.drugdesign.fr.

Set A classification relies on the Structural Classification of Proteins-extended (SCOPe) database (Chandonia *et al.*, 2019; Fox *et al.*, 2014). The lowest hierarchical level—called *Domain* hierarchical level—links the SCOPe database to the Protein Data Bank (PDB) (Berman *et al.*, 2000). The following inclusion procedure was applied on all SCOPe entries. A PDB structure was included if (i) its conformers display the same number of atoms, (iii) it belongs to the *α*, α+β or α/β structural classes of the SCOPe database, (iii) at least four orthologous protein structures exist and satisfy the previous inclusion rules. A total of 211 PDB entries satisfying all these criteria were selected and assigned to 17 classes (following the SCOPe *Protein* hierarchical level). Apart from the *Protein* hierarchical level, the dataset contains sub-classes along two hierarchical sublevels. The *Species* hierarchical level contains 54 classes corresponding to the different species. The *Domain* hierarchical level is composed of 241 classes corresponding to the initial SCOPe classification. For each structure of the dataset, the solvent excluded surface (SES) (Connolly, 1983) was computed using EDTSurf (Xu and Zhang, 2009) with default parameters. EDTSurf outputs triangular meshes stored as .ply file, converted to .off and .pcd formats, required by the different shape comparison methods.

**Set**  B consists in 16 protein structures that were studied in Sael *et al.*, (2008a). The following protein couples in set B display high surface shape similarity and low sequence identity defined by their PDB ID: 1jzn (chain A)—1g1q (chain A), 1 bar (chain A)—1rro, 1ryp (chain B)—1gwz, 1a31—1cy0, 1tbp—1t7p, 1b3t—1adv, 2nwl—2bbh and 2b2i—2cfp.

### 2.2 Shape retrieval methods

In **3D-Surfer**, the protein global surface information is represented with 3D Zernike Descriptors (3DZD), mathematical moment-based invariants of 3D functions (Sael *et al.*, 2008a). The molecular surface of the protein is triangulated using MSROLL (Connolly, 1993) and mapped onto a 3D grid from which 3DZD descriptors are calculated for each protein. The similarity between two given protein surfaces is quantified by the Euclidean distance between their two respective descriptors. 3D-Surfer is only available online (La *et al.*, 2009) and takes a PDB file as an input.

In **PANORAMA** (Papadakis *et al.*, 2010), the panoramic views, i.e. molecular surface maps, are acquired through cylindrical projections of the protein surface. The feature extraction relies on the use of two 2D transforms. Once the descriptor is extracted for each protein surface of the dataset, the Manhattan and the Canberra distances are used to quantify the overall similarity between the two protein surfaces.

**ShapeDNA** is a spectral descriptor (Reuter, 2010; Reuter *et al.*, 2006). The descriptor corresponds to the normalized eigenvalues obtained with the Laplace-Beltrami operator on the protein molecular surface. The similarity between two given protein surfaces is quantified by comparing their spectra using the Euclidean distance.

The **Viewpoint Feature Histogram (VFH)** (Rusu *et al.*, 2010) is a descriptor defined by a histogram of geometrical features (Aldoma *et al.*, 2012). In VFH, a two-components descriptor is calculated from (i) the normal at each point of the discrete surface (i.e. at each vertex of the protein molecular surface mesh triangles) and (ii) the normal of the centroid of the protein molecular surface. VFH is available in the PCL library (Rusu and Cousins, 2011).

### 2.3 Protein structure comparison methods

**CE (Combinatorial Extension)** (Shindyalov and Bourne, 1998) represents proteins as a set of octameric fragments. Each pair of octameric fragments that can be aligned within a given threshold is considered an aligned fragment pair (AFP). CE uses a combinatorial extension algorithm to identify and combine the most similar AFPs between the compared structures. A Z-score is computed for the final alignment using a reference set of alignments (Marti-Renom *et al.*, 2009).

**DeepAlign** (Wang *et al.*, 2013) performs automatic pairwise protein structure alignment using evolutionary relationships and hydrogen-bonding similarity, in addition to spatial proximity of equivalent residues. The scoring function is composed of amino acid mutation score, local substructure substitution potential, hydrogen-bonding similarity and geometric similarity.

**TM-Align** (Zhang and Skolnick, 2005) identifies the best structural alignment between protein pairs independently from their sequences. It first generates optimized residue-to-residue alignment based on structural similarity using heuristic dynamic programming iterations. Then, the scoring function TM-score (Zhang and Skolnick, 2004) is used to scale the structural similarity. TM-score outputs a score *s* in (0,1], where 1 indicates a perfect match between two structures. Output scores below 0.2 usually correspond to unrelated proteins, while those higher than 0.5 assume generally the same fold in SCOP/CATH (Murzin *et al.*, 1995; Orengo *et al.*, 1997).

**lDDT** (local Distance Difference Test) (Mariani *et al.*, 2013) evaluates the fraction of pairwise distances between atoms found in both the reference and the query structures; therefore, lDDT is a superposition-free method well suited to the analysis of flexible protein structures. The distances *D_ij_* between all atom pairs *ij* (from different residues) within 15 Å in the reference structure are computed. The lDDT score is computed as the average of the four fractions of conserved distances between the defined atom pairs *ij* with a growing tolerance of 0.5, 1, 2 and 4 Å, respectively.

**MMLigner** (Collier *et al.*, 2017) relies on the Bayesian framework of Minimum Message Length (MML) criterion. In this framework, the possible 3D superposition of two proteins are considered as representative of their structural relationships expressed as a one-to-one, order-preserving, correspondence between subsets of residues. Therefore, MMLigner generates zero to several possible structural alignments for a pair of input structures. We present here the coverage of the best structural alignment; it is expressed as the fraction of residues being aligned (the smallest protein is taken as the reference).

**KPax** (Ritchie, 2016) is a flexible backbone structural alignment program design to circumvent the limitations of the rigid 3D superposition algorithms. KPax starts by detecting short, local sets of seven residues, then uses dynamic programming to generate an optimal, global alignment using Gaussian functions to score the structural alignments (rigid structural alignment). The residue pairs structurally aligned are then assigned to a segment, and the remaining residues are considered for a new alignment step until no further residue sets of seven residues can be aligned. The resulting structural alignments are evaluated using the M-Score (Ritchie, 2016), that scale from 0 (no alignment) to 1 (perfect structural alignment).

### 2.4 Shape retrieval performance evaluation

The performance in retrieval of each method was evaluated using Precision-Recall curves, Nearest Neighbor (NN), First-tier (FT), Second-tier (ST) and Mean Average Precision (MAP). The Precision-Recall plot draws the recall *R* as a function of the precision *P*. Precision *P* is the ratio of targets from class *C* retrieved within all objects attributed to class *C*, while recall *R* represents the ratio of retrieved targets from class *C* compared to |C|, the size of class *C*. NN, FT and ST check the ratio of targets successfully attributed to the class *C*. For NN, only the top-ranked match is considered. For FT and ST, the |C|−1 and 2|C|−1 first matches are considered, respectively. The MAP is the Mean Average Precision for each query, which is the average of all precision values computed when each relevant target is retrieved.

### 2.5 Runtime

To evaluate the computation time of the shape retrieval methods, we considered the sum of the runtimes required to compute (i) the method’s descriptor for the largest SES, (ii) the method’s descriptor for the smallest SES and (iii) the distance between two SES. All calculations were performed on an Intel Core i7-6700HQ CPU@2.60 GHz with 32 GB of RAM.

### 2.6 Identifying distant surficial homologs

In order to identify hits with low sequence identity but similar surface shapes (*i.e.* distant surficial homologs), we compared the dissimilarity matrices to a sequence aligning matrix. Clustal Omega (Sievers *et al.*, 2011) was used to align all the sequences of set A. A homology matrix $H_{N,N}=5298$ was constructed, such as $H_{\left[i,j\right]},\;(i,j)\in {\left[[1,5298]\right]}^{2}$ enrolls the sequence identity ratio between protein *i* and protein *j*. Then, the dissimilarity matrix output $M_{N}^{k},\;k=1,2,3,4$, of each method was normalized, and we computed $H+M_{N}^{k}$. For each method, $\min_{j}H+M_{N}^{k}$ represents the protein target *j* combining the least sequence identity and the highest shape similarity for protein query *i*. Afterwards, $\min_{j}H+M_{N}^{k}$ for each method *k* was compared. If at least two methods *k*_1_ and *k*_2_ bring the same $\min_{j}H+M_{N}^{k}$, the protein pair *i* and *j* are considered distant surficial homologs.

## 3 Results

First, we present and compare the performance in retrieval of the shape retrieval methods (3D-Surfer, PANORAMA, ShapeDNA, VFH) on the hierarchical protein shapes set A. Then, we illustrate their performance (i) on the calmodulin that displays very large conformational changes and (ii) in identifying distant surficial homologs (low sequence identity with high shape similarity). The performance of widely used structure comparison methods (CE, DeepAlign, TM-Align, lDDT) is presented as a reference.

### 3.1 Protein shape retrieval

**Fig. 1.**
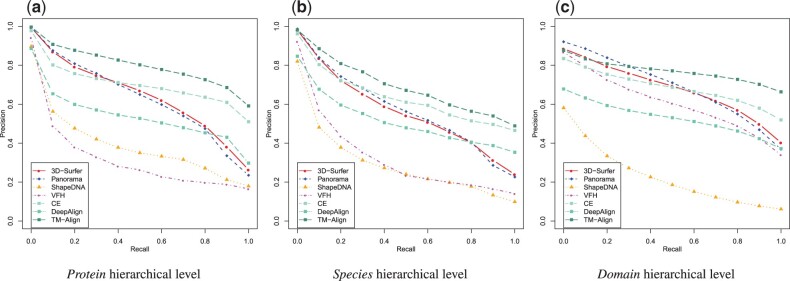
Precision-Recall plots for the shape retrieval methods (3D-Surfer, PANORAMA, ShapeDNA, VFH) and structure comparison methods (CE, DeepAlign, TM-Align) over the different hierarchical levels of set A

Table 1 summarizes the quantitative statistics values for each method on the three hierarchical levels, *Protein*, *Species* and *Domain*. Figure 1 presents the precision-recall curves for each method and each hierarchical level.

**Table 1.** Retrieval statistics computed for each method and each hierarchical level of set A

Method	Hierarchy	NN	FT	ST	MAP
3D-Surfer	Protein	**0.993**	0.591	0.726	0.653
	Species	**0.979**	0.566	0.656	0.593
	Domain	0.792	0.689	0.840	0.721
PANORAMA	Protein	0.988	0.576	0.716	0.630
	Species	0.977	0.540	0.644	0.566
	Domain	**0.806**	0.647	0.792	0.684
Shape-DNA	Protein	0.816	0.348	0.534	0.367
	Species	0.710	0.273	0.420	0.270
	Domain	0.415	0.236	0.322	0.213
VFH	Protein	0.900	0.271	0.443	0.287
	Species	0.880	0.289	0.414	0.306
	Domain	0.788	0.573	0.689	0.599
CE	Protein	0.953	0.675	0.829	0.696
	Species	0.940	0.598	0.693	0.625
	Domain	0.740	0.648	0.781	0.676
DeepAlign	Protein	0.678	0.513	0.679	0.517
	Species	0.668	0.486	0.623	0.489
	Domain	0.447	0.488	0.666	0.500
TM-Align	Protein	0.991	**0.749**	**0.897**	**0.793**
	Species	0.973	**0.648**	**0.747**	**0.685**
	Domain	0.797	**0.736**	**0.859**	**0.758**

*Note*: Bold numbers represent the best value in each category

#### 3.1.1 *Protein* hierarchical level

The precision-recall curves in Figure 1a show similar performance between 3D-Surfer and PANORAMA, and between ShapeDNA and VFH. The high performance of 3D-Surfer and PANORAMA are corroborated by Table 1, where the NN statistics display values greater than 0.98 and MAP statistics greater than 0.5 for both methods. For FT and ST statistics, 3D-Surfer and PANORAMA surpass ShapeDNA and VFH as well. 3D-Surfer and PANORAMA outperform the structure comparison methods CE and DeepAlign for recall values below 0.5. In particular, 3D-Surfer displays the best NN followed by TM-Align, PANORAMA, CE, VFH, ShapeDNA and DeepAlign, respectively.

#### 3.1.2 *Species* hierarchical level

As the hierarchical level goes down—from the *Protein* hierarchical level to the *Species* hierarchical level, the orthologous proteins are separated into disjoint classes. The overall performance decrease for the shape retrieval methods, except for VFH in FT and MAP (Table 1, Fig. 1b). For the NN performance metric, as previously observed in the *Protein* hierarchical level, the best performance are associated with 3D-Surfer, followed by PANORAMA, TM-Align, CE, VFH, ShapeDNA and DeepAlign, respectively.

#### 3.1.3 *Domain* hierarchical level

In this hierarchical level, the classes are the least populated (from 2 to 160 protein objects). We observe a flattening of the precision-recall curves (Fig. 1c) for the shape retrieval methods, except for ShapeDNA whose performance are in decay with respect to the *Species* and the *Protein* hierarchical level (Table 1). Regarding the other methods, only the NN statistic drops down compared to the higher hierarchical levels, with PANORAMA displaying the best value, followed by TM-Align, 3D-Surfer, VFH, CE, DeepAlign and ShapeDNA, respectively. Except for ShapeDNA, all methods displayed increased performance in retrieval on this level in the FT, ST and MAP compared to the *Species* hierarchical level.

#### 3.1.4 Runtime

The runtimes are presented in Table 2, with a distinction between the runtimes for the descriptors calculation and the runtimes for the descriptors comparison. Results for 3D-Surfer are not reported since it is a full web-service. In total runtime, we observe that VFH is the fastest, followed by PANORAMA and ShapeDNA, respectively. The structure comparison methods mean computation times for CE, DeepAlign and TM-Align are 2, 0.2 and 0.1 s, respectively.

**Table 2.** Runtimes (in seconds) to compute the descriptor for the largest and the smallest SES of the dataset and to compute the distance between two SES using the evaluated shape retrieval methods

Method	PANORAMA	Shape-DNA	VFH
Largest SES	5.05	19.05	2.31
Smallest SES	1.04	2.98	0.44
Distance	0.27	0.02	0.03
Total	6.36	22.05	2.78

*Note*: For information, EDTSurf takes 10.9 s and 1.85 s to compute the largest and smallest SES, respectively.

### 3.2 Proteins displaying large conformational changes: the calmodulin case

Proteins are dynamical objects that may undergo large structural, conformational changes. In such cases, usual rigid-body superposition-based comparison methods display some well-known weaknesses: only C*α* (or backbone) atoms are taken into consideration; they are sensitive to the changes of orientation between the domains of multi-domains proteins; models bearing unrealistic structural features (steric clash, for instance) are not penalized (Aloy *et al.*, 2003; Shi *et al.*, 2009). In set A, a few classes displayed conformational variability; we focus here on the specific example of the *Xenopus laevis* calmodulin, whose structure undergoes a large and ample re-arrangement of its domains.

***Xenopus laevis* calmodulin** is composed of two domains linked by a three-residue coil that allows an ample motion of one domain with respect to the other (Supplementary Table S1), resulting in very different conformations (pdb entry 1dmo, chain A, 30 conformers). In order to investigate whether the selected shape retrieval methods are able to retrieve these high-amplitude non-rigid transformations, we enumerated the number of 1dmo (chain A) conformers retrieved for each query within the first 30 retrieved shapes (Table 3, Supplementary Fig. S1). Shape retrieval methods retrieved on average at least 7.13 conformers within the top 30 for each of the 30 queries (9.60, 10.70, 7.13 and 14.46 for 3D-Surfer, PANORAMA, ShapeDNA and VFH, respectively). Structure comparison methods retrieved on average less than 5.86 conformers for each query (2.3, 5.86 and 2.03 for CE, DeepAlign and TM-Align, respectively). To complement the structure comparison methods that are all superposition-based, we added a reference superposition-free structure comparison method, lDDT (Mariani *et al.*, 2013). On this task, lDDT retrieved on average 21.87 conformers for each query. A more detailed analysis of these results showed that all proteins retrieved by lDDT within the top 30 are conformers either from 1dmo or 1f70, which corresponds to the N-terminal domain of the *Xenopus laevis* calmodulin.

**Table 3.** Mean number of conformations retrieved within the top 30 results for the class 1dmo (chain A) with the different shape retrieval (top) and structure comparison (bottom) methods

	1dmo (chain A)	
	Mean	SD
3D-Surfer	9.60	2.45
PANORAMA	10.70	3.41
ShapeDNA	7.13	3.28
VFH	14.46	5.28
CE	1.91	2.30
DeepAlign	5.86	2.55
TM-Align	2.03	2.05
lDDT	21.87	2.51

SD, standard deviation.

### 3.3 Identifying distant surficial homologs

**Fig. 2.**
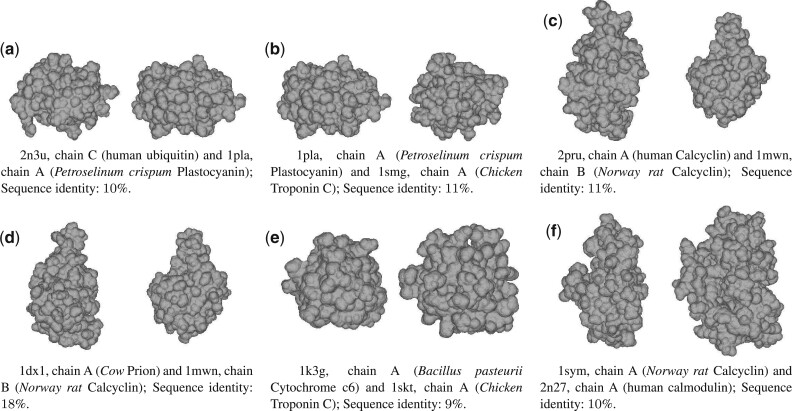
Distant surficial homologs identified using the shape similarity search methods

Shape retrieval methods allow to compare protein structures regardless of their sequences, secondary structures or fold. We identified in set A, 6 pairs of protein shapes (Fig. 2) sharing up to 19% sequence identity but displaying similar surface shapes (*i.e.* distant surficial homologs). From the biological function point of view, 3 out of the 6 pairs share different biological functions. The pairs *b*, *f* and *e*, respectively associate a calcium-binding protein and an electron binding protein; a calcium-binding protein and a metal-binding protein; a metal-binding protein and an electron transport protein.

We compared the distances obtained for these protein pairs with the distances obtained within distant surficial homologs retrieved from the literature (Sael *et al.*, 2008a) (set B, Table 4). Using the maximum distances observed for the selected protein pairs from set A as a similarity threshold for the protein pairs in set B, 1, 0, 3 and 7 out of the 8 pairs from set B were identified by 3D-Surfer, PANORAMA, ShapeDNA and VFH, respectively. On the same task, considering 2 Å as a typical similarity threshold for protein structures (Samudrala and Levitt, 2002), CE retrieved zero pair and DeepAlign one pair. One out of the eight pairs was considered to be similar using TM-Align [TM-score > 0.5 (Zhang and Skolnick, 2004)].

**Table 4.** Distance values and scores for the protein couples of set B (Sael *et al.*, 2008a) according to the shape similarity search and the structure comparison methods

Method	1jzn (chain A)	1bar (chain A)	1ryp (chain B)	1a31	1tbp	1b3t	2nwl	2b2i
	1g1q (chain A)	1rro	1gwz	1cy0	1t7p	1adv	2bbh	2cfp
3D-Surfer (Euclidian distance)	7.60	8.62	11.88	4.72	7.46	8.07	7.57	5.80
PANORAMA (composite distance)	0.0263	0.0255	0.0251	0.0278	0.0237	0.0264	0.0247	0.0230
Shape-DNA (Euclidian distance)	0.59	0.60	2.19	0.51	1.74	2.01	1.93	1.42
VFH (Euclidian distance)	90.12	70.67	646.74	167.55	76.42	174.30	150.02	69.05
CE (RMSD, Å)	2.48	5.48	6.54	6.47	3.35	5.88	7.77	5.04
DeepAlign (RMSD, Å)	1.97	5.07	5.65	7.01	3.51	4.30	2.83	6.05
TM-Align (TM-score)	0.65	0.30	0.26	0.24	0.14	0.21	0.19	0.31
MMLigner (coverage)	0.28	0	0	0	0	0	0.29	0.20
KPax (M-score)	0.19	0.17	0.11	0.07	0.08	0.12	0.17	0.13
3DZD^a^ (Euclidian distance)	52.6	12.6	12.7	5.58	7.25	7.65	6.04	7.28
CE^a^ (RMSD, Å)	2	6.7	5	6.3	4.9	6.7	8.1	4.9
SeqID^a^ (Sequence identity, %)	23.5%	3.6%	9.7%	5.8%	2%	9%	5.7%	7.8%

*Note*: For 3D-Surfer, PANORAMA, Shape-DNA, VFH and 3DZD: the lower the distance, the higher the similarity. TM-Align (TM-score), MMLigner (coverage) and KPAX (M-score) values range from 0 (no similarity) to 1 (ideal similarity). For CE and DeepAlign, the lower the values, the higher the similarity. For SeqID, the higher the percentage, the higher the similarity. Only RMSD (from CE and DeepAlign) values are directly comparable. The chain has been specified when different chains are available in the PDB file.

a Reproduced from Sael *et al.* (2008a).

## 4 Discussion

### 4.1 Protein shape retrieval

The performance of the shape retrieval methods were evaluated on the hierarchical dataset of protein shapes (set A) that is exclusively composed of NMR structures. We decided to use NMR structures because they represent the biological dynamics of proteins with different models based on constraints derived from experimental data (Marion, 2013; Mittermaier and Kay, 2009). The inclusion of these different models allowed to evaluate the influence of the conformational variability of each protein in our dataset on the performance of the evaluated methods in retrieval. It is to note that the variability of side chain conformations displayed a low influence on the overall performance of the evaluated methods in retrieval (Supplementary Fig. S2).

This evaluation highlighted the high performance of 3D-Surfer, the only shape retrieval method evaluated in the present work originally designed to compare protein shapes. Its performance was tightly followed by PANORAMA, a shape retrieval method that was never applied to protein shapes to date.

Despite displaying high performance in different non-molecular shapes benchmarks (Li *et al.*, 2015; Lian *et al.*, 2013; 2015; Li and Hamza, 2014), ShapeDNA and VFH were outperformed by 3D-Surfer and PANORAMA. This suggests that the shape representation used in ShapeDNA and VFH could be less adapted to protein shapes and highlights the complexity of molecular shapes compared to the smoother shapes of the 3D objects classically used in computer vision (furniture, buildings, animals, human faces, …). Proteins and molecular objects in general are considered feature-less compared to classical 3D-objects that usually display easily extractable and matchable features (wheels, ears, nose, legs, …) (Langenfeld *et al.*, 2019). The extraction of standard 3D descriptors for a homogeneous surface such as a protein surface could result in ambiguous correspondences unless the descriptor is able to scale-up with a higher level of detail, notably in the *Protein* and the *Species* hierarchical level. In particular, the lower performance of VFH can be explained by the use of normals to obtain the descriptor. The roughness of protein surfaces adds angular noise to the normals of the molecular surface. For similar reasons, since ShapeDNA is a descriptor defined by the geometry, the performance of ShapeDNA can be explained by the uneven surfaces of proteins. On the contrary, the high performance of 3D-surfer can be explained by the fact that Zernike moments are based on spherical harmonics. Spherical harmonics represent a basis function on the surface of a sphere. This descriptor is particularly adapted to globular proteins that mainly compose the datasets used in this study. PANORAMA describes the cylindrical projection by a discrete transform which is a sum of functions defined on a circle. These functions are well-fitted to describe the globular shape of the proteins composing the datasets projected on a cylinder as well.

At the *Species* hierarchical level, orthologous proteins are separated in different classes. Most shape retrieval methods classified orthologous proteins within the same class, resulting in a loss of performance in retrieval compared to the *Protein* hierarchical level. Similarly, at higher recall values, the best structure-based methods still outperform the shape-based methods (Fig. 1 and Table 1). These results point at potential ways for future improvements such as taking additional molecular surface features (electrostatics, hydrophobicity, …) into consideration (Gainza *et al.*, 2020). Concerning the computational costs associated with the shape retrieval methods, it is important to note that in application cases such as large databases screening, the speed of calculation of the distance between the methods descriptors is a key factor contrarily to the cost associated to the calculation of the descriptor that can be performed only once per object and stored for future use. In this regard, Shape-DNA, VFH and 3D-Surfer are extremely satisfying since the distance computation takes respectively, 20, 30 and 60 ms. PANORAMA can still be usable for screening large databases with a distance computational cost of 270 ms.

### 4.2 Proteins displaying large conformational changes: the calmodulin case

Methods able to retrieve the different conformational states of a given protein can be very useful, notably in cryo-Electron Microscopy (cryo-EM) and cryo-Electron Tomography (cryo-ET) where detected macromolecular shapes can be identified using shape retrieval methods (Han *et al.*, 2019). We illustrated the adaptability of the different shape retrieval methods to protein conformational changes using the example of the *Xenopus laevis* calmodulin that displays very ample motions of its domains (Table 5).

**Table 5.** Illustration of the conformational changes of the Xenopus calmodulin (PDB ID 1dmo, chain A)

Conformer	17	12	18	22	24
lDDT	–	0.7299	0.7396	0.7701	0.7748

*Note*: The model’s numbers are indicated below each structure shape. Chain A of model 17 of PDB ID 1dmo was taken as reference to compute the lDDT score (Mariani *et al.*, 2013).

Shape retrieval methods outperformed superposition-based structure comparison methods, but were outperformed by lDDT, a reference superposition-free structure comparison method. However, it is to note that lDDT and the other superposition-free structure comparison methods such as CAD-score (Olechnovič *et al.*, 2012), KPAX (Ritchie, 2016) or MMLigner (Collier *et al.*, 2017) are limited to the comparison of proteins of partially similar topology to be efficient. A typical example is the comparison of a predicted structure to a reference experimental structure (Mariani *et al.*, 2013) like in the CASP-CAPRI experiment (Lensink *et al.*, 2019). Otherwise, in the case of a comparison with distant surficial homologs, they will likely fail to produce a meaningful result since they are not primarily designed for this task (Mariani *et al.*, 2013) (Table 4). On the contrary, the best performing method from the computer vision field, VFH, was designed to track the mobility of objects on camera snapshots over time (Rusu and Cousins, 2011). This highlights one of the advantages of comparing proteins through their molecular surface shapes since the protein molecular surface representation abstracts the layers of complexity beneath the surface, *i.e.* the fold and secondary structures encoded in the backbone atoms 3D coordinates. The poor performance of TM-Align in this task could be explained by its residue to residue optimization that may have failed with the large motion of the second domain of the *Xenopus laevis* calmodulin.

These results shed lights on the versatility of shape retrieval methods. While structure-based methods require either rigid-body superposition (CE, TM-Align…) or a high similarity between the objects to be compared (lDDT, CAD-score…), shape-based methods, due to the abstract protein molecular surface representation, may be better suited to the blind classification of large datasets including highly heterogeneous protein structures (Guzenko *et al.*, 2020; Han *et al.*, 2019; Mavridis and Ritchie, 2010), such as a screening of the Protein Data Bank (Berman *et al.*, 2000). Further work dedicated to this specific task would be beneficial to the community.

### 4.3 Identifying distant surficial homologs

The protein molecular surface representation is an abstraction of the primary, secondary and tertiary structure representations. Functionally related proteins often share similar surface properties despite a low sequence and/or backbone conformation similarity (Han *et al.*, 2019; Sael *et al.*, 2008a). Identifying distant surficial homologs *i.e.* proteins with similar molecular surface shapes and low sequence identity, is of a major interest. It underlines the usefulness of shape retrieval methods and beyond, tackling protein structure comparison through their molecular surface shape instead of their backbone orientation, especially when structural methods fail to identify such similarity. Shape retrieval methods could be used to identify proteins with similar molecular surfaces despite a low sequence identity which could be beneficial to the protein structure prediction community, notably in threading where folds could be enriched with surface shapes. Similarly, these methods may be beneficial to the protein structure classification where reference databases still require manual intervention from human experts. With this in mind, shape-based methods may enrich the pool of methods available for protein comparison able to retrieve proteins with similar shape but different topologies (Mavridis and Ritchie, 2010; Wen *et al.*, 2020). This could also be useful for identifying possible interacting partners (Gainza *et al.*, 2020) since molecular shape plays a crucial role in binding (Levieux *et al.*, 2014; Pawlowski and Godzik, 2001; Shulman-Peleg *et al.*, 2004). Shape retrieval methods could then be used for creating a structural classification of proteins based on their surfaces (Han *et al.*, 2019; Sasin *et al.*, 2007), rather than evolutionary distances or fold categories as in SCOP (Murzin *et al.*, 1995) or CATH (Orengo *et al.*, 1997) opening the possibility to extend the protein structure–function paradigm toward a protein structure-surface(s)-function paradigm.

Here, we extended set B by using a consensus of the shape retrieval methods evaluated in this study to screen set A. Six protein pairs were identified in set A displaying similar surface shapes with sequence identity below 19% (Table 6 and Fig. 2). These protein pairs from sets A and B could constitute a useful resource for the evaluation of the performance of future shape retrieval methods to identify distant surficial homologs.

**Table 6.** Output values for each method on the identified distant surficial homologs couples illustrated in Figure 2

Protein couple	a	b	c	d	e	f
3D-Surfer	4.26	5.26	3.87	3.37	4.02	3.57
PANORAMA	0.0219	0.0227	0.0217	0.0213	0.0221	0.0222
Shape-DNA	1.09	0.22	0.57	0.73	0.37	0.79
VFH	180.05	57.81	44.87	33.22	79.14	31.41
CE	5.75	5.54	4.76	4.94	5.36	3.35
DeepAlign	4.46	3.21	3.55	3.43	3.24	3.51
TM-Align	0.31	0.29	0.24	0.27	0.23	0.79
MMLigner	0.36	0	0.79	0.53	0.72	0.60
KPax	0.35	0.37	0.44	0.35	0.31	0.34
SeqID	10.11%	11.23%	11.26%	18.42%	9.85%	10.14%

*Note*: For 3D-Surfer, PANORAMA, Shape-DNA, VFH and 3DZD: the lower the distance, the higher the similarity. TM-Align (TM-score), MMLigner (coverage) and KPAX (M-score) values range from 0 (no similarity) to 1 (ideal similarity). For CE and DeepAlign, the lower the values, the higher the similarity. For SeqID, the higher the percentage, the higher the similarity. Only RMSD (from CE and DeepAlign) values are directly comparable.

## 5 Conclusion

In this work, we evaluated the performance of four shape retrieval methods from the computer vision field (3D-Surfer, PANORAMA, Shape-DNA and VFH) on a protein shapes dataset. On this dataset, 3D-Surfer and PANORAMA outperformed Shape-DNA and VFH. On a selected example displaying large conformational changes (calmodulin), all shape retrieval methods displayed a reasonable performance in recognizing their different conformations within the dataset.

Different structure comparison methods were used as a reference in this study (CE, DeepAlign and TM-Align). TM-Align slightly outperformed shape retrieval methods in the retrieval task, but failed in tracking the large conformational changes of the calmodulin. For the calmodulin case, the superposition-free structure comparison method lDDT outperformed all the other evaluated methods in identifying the different conformers of calmodulin. We also identified six pairs of distant surficial homologs that could be used for future studies on protein surficial similarity search. Finally, shape retrieval methods were associated with larger computational costs compared to classical structural alignment methods but this additional cost is still compatible with the treatment of large structural datasets. Geometric learning methods could be beneficial here since their computational cost seems to be lower.

This work confirms the interest of protein molecular shape as a higher-level description of the protein structure that (i) abstracts the underlying protein sequence, structure or fold, (ii) allows the use of shape retrieval methods to screen large databases of protein structures to identify surficial homologs and possible interacting partners, (iii) opens an extension of the protein structure–function paradigm toward a protein structure-surface(s)-function paradigm.

## Supplementary Material

btab511_Supplementary_Data
