# Supplementary material for: Comparative evaluation of shape retrieval methods on macromolecular surfaces: an application of computer vision methods in structural bioinformatics
Source: Bioinformatics. 2021 Jul 11;37(23):4375–82. doi: 10.1093/bioinformatics/btab511 (PMC8652110; doi:10.1093/bioinformatics/btab511)
Supplement: btab511_Supplementary_Data [file btab511_supplementary_data.docx]

Supplementary Information for:

**Comparative Evaluation of Shape Retrieval Methods on Macromolecular Surfaces: An Application of Computer Vision Methods in Structural Bioinformatics**

Mickael Machat ^1^, Florent Langenfeld ^1^, Daniela Craciun ^1^, Léa Sirugue ^1^, Taoufik Labib ^1^, Nathalie Lagarde^1^, Maxime Maria ^2,1^ and Matthieu Montes ^1,∗^

1 Laboratoire GBCM, EA 7528, Conservatoire National des Arts et Métiers, Hesam université, 2 rue Conté, 75003 Paris, France

2 Laboratoire XLIM, UMR CNRS 7252, Université de Limoges, 123 avenue Albert Thomas, 87000 Limoges, France

∗To whom correspondence should be addressed

**Supplementary Table S1**.

Illustration of the conformational changes of the Xenopus calmodulin (PDB ID 1dmo, chain A) with the conformers presented in Figure 3. The lDDT score [1] was computed using the conformer 1dmo_A_17 as reference.

*corresponds to the angle value between the principal axes of the two moving domains of the conformer.

** corresponds to the torsion angle between the principal plans of the two moving domains of the conformer. The angle and torsion angle were computed using LOOS [2].

| Conformer | 17 | 12 | 18 | 22 | 24 |
| --- | --- | --- | --- | --- | --- |
| LDDT Score | - | 0.7299 | 0.7396 | 0.7701 | 0.7748 |
| Angle * | 61.09 | 25.34 | 56.9 | 49.68 | 150.52 |
| Torsion Angle ** | -39.7 | 15.01 | 72.29 | -55.12 | -162.26 |

**Supplementary Figure S1**.

First-tier values for the classe 1dmo_A. The individual first-tier values are shown for each method: each point corresponds to the first-tier value computed with a given shape taken as query. Each box covers the inter-quartile range, with the median indicated by an horizontal line in the middle of the box. The whiskers extend to 1.5 of the interquartile range


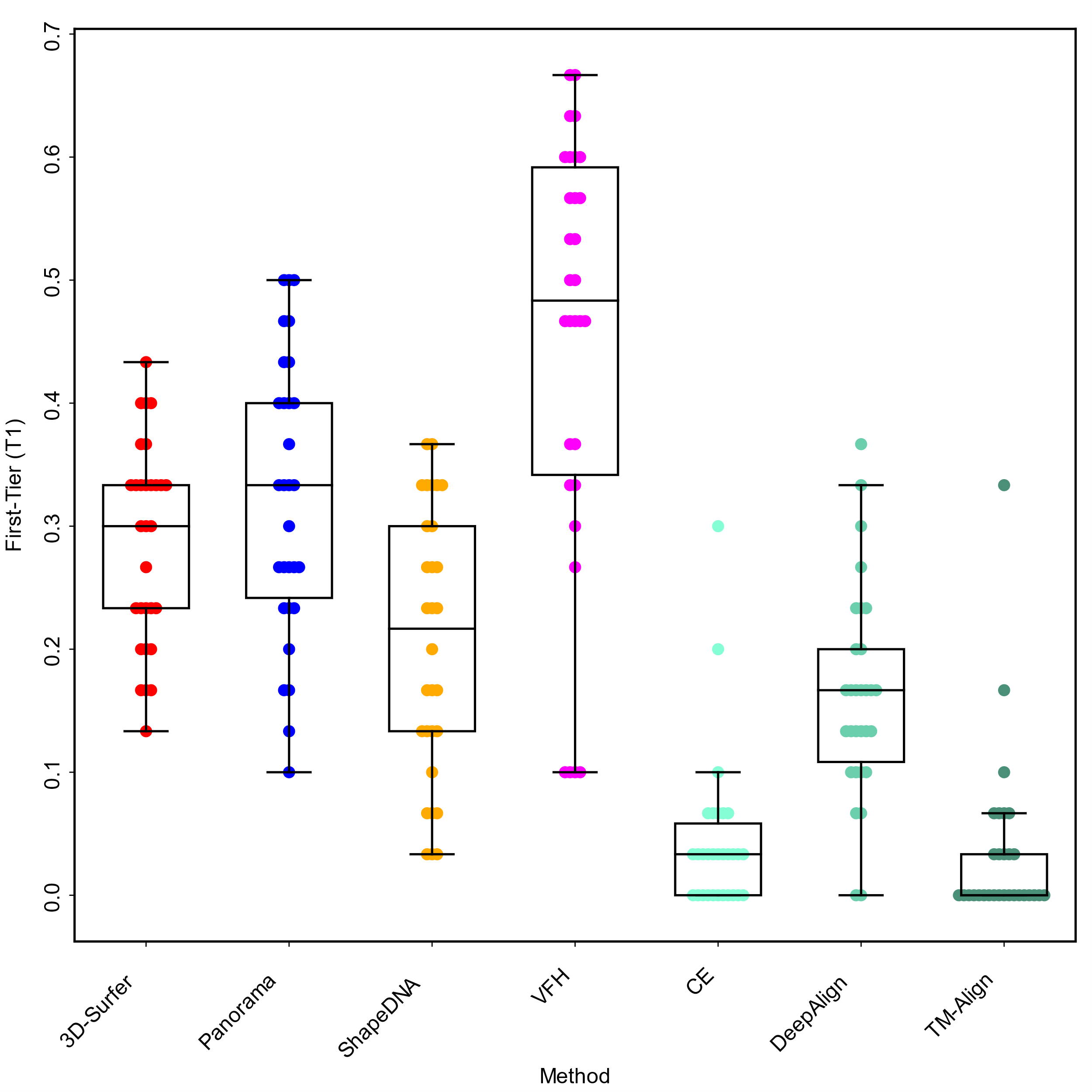


**Supplementary Figure S2**.

Dissimilarity matrices obtained from the four studied shape retrieval methods on 120-target dataset including an outlier protein NMR set:

1dmo_pdb: PDB NMR set 1dmo (chain A), 30 conformers

1dmo_nosc: PDB NMR set 1dmo (chain A) without side chain, 30 conformers

1dmo_scwrl: PDB NMR set 1dmo (chain A) with side chains built with SCWRL4, 30 conformers

outlier: PDB NMR set 1pla (chain A), 30 conformers

We performed additional analysis to study the sensitivity of the shape-retrieval methods to the conformations of the side chains on the very flexible example of the *Xenopus Levis* calmodulin, PDB code 1dmo. For each conformation, we compared the performance of the shape retrieval methods obtained with (1) the initial NMR structures, (2) the NMR structures without side chains and (3) the NMR structures with rebuilt side chains obtained from scwrl4 (see Figure below). Very similar performances were obtained with the sidechain modalities (original or reconstructed), showing that the global shape of the proteins is not affected by the conformations of the side chains.

We can thus conclude that shape-retrieval methods aren’t sensitive to the conformations of the side chains.


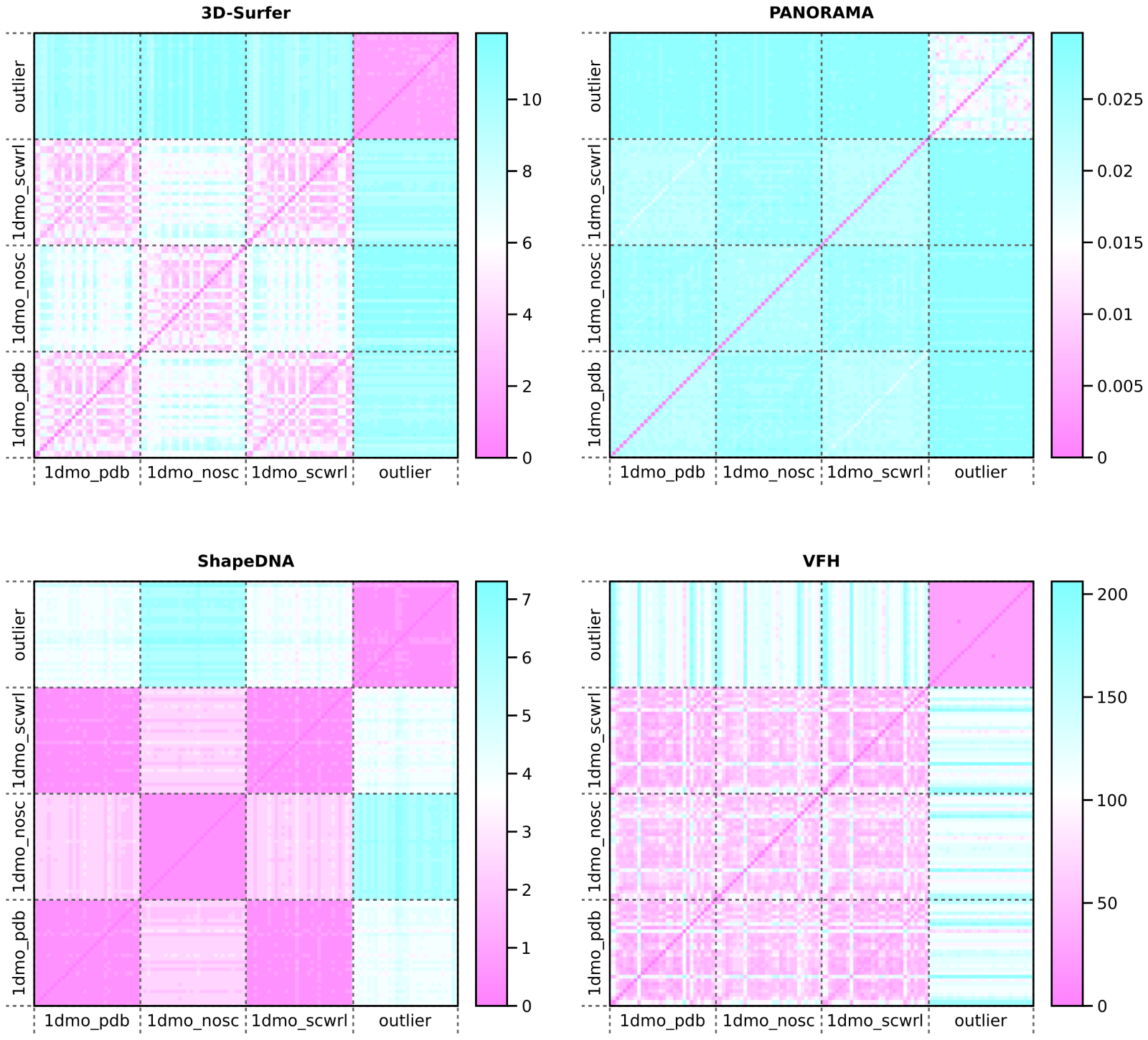


**References**

1. Valerio Mariani, Marco Biasini, Alessandro Barbato, and Torsten Schwede. lddt: a local superposition-free score for comparing protein structures and models using distance difference tests. Bioinformatics, 29(21):2722–2728, 2013.
2. Tod D Romo, Nicholas Leioatts, and Alan Grossfield. Lightweight object oriented structure analysis: tools for building tools to analyze molecular dynamics simulations. Journal of computational chemistry, 35(32):2305–2318, 2014.
